# Primary care service use by end-of-life cancer patients: a nationwide population-based cohort study in the United Kingdom

**DOI:** 10.1186/s12875-020-01127-8

**Published:** 2020-04-29

**Authors:** Wei Gao, Martin Gulliford, Myfanwy Morgan, Irene J. Higginson

**Affiliations:** 1 Department of Palliative Care, Policy and Rehabilitation, Faculty of Life Sciences & Medicine, Cicely Saunders Institute, King’s College London, London, UK; 2 Faculty of Life Sciences & Medicine, School of Division of Primary Care & Public Health Sciences, King’s College London, London, UK; 3 Faculty of Life Sciences & Medicine, Institute of Pharmaceutical Science, King’s College London, London, UK

**Keywords:** General practice, End of life care, Palliative care, Cancer, Healthcare service use, Healthcare access inequalities

## Abstract

**Background:**

End of life (EoL) care becomes more complex and increasingly takes place in the community, but there is little data on the use of general practice (GP) services to guide care improvement. This study aims to determine the trends and factors associated with GP consultation, prescribing and referral to other care services amongst cancer patients in the last year of life.

**Methods:**

A retrospective cohort study of cancer patients who died in 2000–2014, based on routinely collected primary care data (the Clinical Practice Research DataLink, CPRD) covering a representative sample of the population in the United Kingdom. Outcome variables were number of GP consultations (primary), number of prescriptions and referral to other care services (yes vs no) in the last year of life. Explanatory variables included socio-demographics, clinical characteristics and the status of palliative care needs recognised or not. The association between outcome and explanatory variables were evaluated using multiple-adjusted risk ratio (aRR).

**Results:**

Of 68,523 terminal cancer patients, 70% were aged 70+, 75% had comorbidities and 45.5% had palliative care needs recognised. In the last year of life, a typical cancer patient had 43 GP consultations (Standard deviation (SD): 31.7; total = 3,031,734), 71.5 prescriptions (SD: 68.0; total = 5,074,178), and 21(SD: 13.0) different drugs; 58.0% of patients had at least one referral covering all main clinical specialities. More comorbid conditions, prostate cancer and having palliative care needs recognised were associated with more primary care consultations, more prescriptions and a higher chance of referral (aRRs 1.07–2.03). Increasing age was related to fewer consultations (aRRs 0.77–0.96), less prescriptions (aRR 1.09–1.44), and a higher chance of referral (aRRs 1.08–1.16) but less likely to have palliative care needs recognised (aRRs 0.53–0.89).

**Conclusions:**

GPs are very involved in end of life care of cancer patients, most of whom having complex care needs, i.e. older age, comorbidity and polypharmacy. This highlights the importance of enhancing primary palliative care skills among GPs and the imperative of greater integration of primary care with other healthcare professionals including oncologists, palliative care specialists, geriatricians and pharmacists. Research into the potential of deprescribing is warranted. Older patients have poorer access to both primary care and palliative care need to be addressed in future practices.

## Background

End of life (EoL) care refers to care that is provided and delivered in the last year of life, which accounts for approximately 10 to 20% of healthcare costs [1]. As we live longer with more comorbidities, the complexity of need at the end of life is bound to increase. This coupled with the projected rise in numbers of people dying with cancer over the next two decades and people’s preference to be cared for and die at home or in a home-like environment [2–4], highlights the importance that providing quality care and support to people with terminal cancer in the community care settings. It is also a policy priority for health care in many countries [5–7]. However, in the United Kingdom, nearly half of people with cancer die in hospitals and just a quarter die at home which is low compared with other high-income countries like the United States, the Netherlands, or Italy [8].

General practitioners (GPs, or family/primary care doctors), the primary care providers in the community, play an important role in EoL care which include, for example, care planning and coordination, and providing continuity of care. However, even in the United Kingdom (UK), where the quality of death ranked the best in the world [9], the EoL care is far from optimal. In a recent survey of bereaved people, one in four rated the overall quality of EoL care for their relatives as poor or fair. Care quality from GPs came as the second worst service provider among the 7 providers surveyed, with 30% poor or fair ratings, next to urgent care [10]. As GPs not only deliver end of life care but also act as a gatekeeper for patients to access other health care services, how patients interact with GP services therefore has a direct impact on their access to other healthcare services and with implications for health outcomes [11].

However, although there is limited data on the service use of primary care by patients towards the end of life, existing studies indicate that there are considerable cross-country variations in both the quality and intensity of care, as well as room for improvement [12–15]. This identifies a need for more country-specific data on the use of primary care services by end of life cancer patients as an essential step to understanding and identifying gaps in service provision. Furthermore, palliative care is a care provided to seriously ill patients that aims to improve quality of life and reduce suffering. It is a holistic approach of care and deemed more appropriate for patients at the end of life. Primary care providers are particularly well positioned to deliver primary palliative care, due to the longitudinal nature of their relationships with patients and families, delivering care in people’s most preferred care settings [2–4]. It is unclear if a patient’s palliative care needs known to their GPs plays a role in the primary care service use of the patient.

This study based on real-world data from primary care in the UK aimed to: 1) describe the patterns of primary care service use among cancer patients in the last year of life, 2) evaluate the role of recognising patients’ palliative care needs in primary care service use, and 3) identify patient characteristics associated with the use of primary care services and with patients palliative care needs recognised by their GPs. We chose to focus on cancer as in the UK it is a condition for which people are more likely to die in a community care setting than for other conditions [8]. GPs therefore have more involvement in the care of these patients towards end of life, which is captured in the primary care database.

## Method

### Study design and setting

A nationwide, population based retrospective cohort study in UK primary care, 2000–2014.

### Data sources

The data were extracted from the Clinical Practice Research Datalink (CPRD) – one of the world’s largest longitudinal primary care databases [16]. By the mid-year of 2013, the CPRD contained anonymized primary care health records for 4.4 million active (alive, currently registered) patients from 7.2% (*n* = 674) of all UK general practices. Patients are broadly representative of the UK general population in terms of age, sex and ethnicity. The CPRD contains data generated during the process of health care in general practices, with demographics, and longitudinal information on clinical aspects (e.g. diagnoses, symptoms, comorbidity), GP contacts, prescriptions, management and referrals and so on. CPRD has been widely applied to health service research [16].

### Patient cohort

The inclusion criteria were all patients who: 1) were diagnosed with a common cancer, as ascertained by Read codes [17] (Lung – B22; Colorectal – B13, B14, B1z0.11; Female breast – B34, B36..00; Prostate – B46); and 2) had at least 6 months of registration with the practice before the cancer diagnosis; and 3) died between 01/01/2000 and 30/04/2014 inclusive; and 4) were registered at a general practice with acceptable data quality.

### Study variables

Outcome variables were the service use in the last 3 months of life in three main categories: a) GP consultations (primary), b) medicines prescribed (using the CPRD unique product codes selected by GPs) and c) referral to secondary care or other care services. We included consultations involving patient contacts, either through face-to-face or telephone, irrespective of where the consultations taking place. We excluded the palliative care referral as this was used to identify patients having palliative care needs recognised (detailed as below).

The explanatory variables were: 1) socio-demographics – age (< 50, 50–59, 60–69, 70–79, 80–89, 90+), gender (female, male), year of death, the region where the patient registered general practice was based; 2) clinical variables – cancer site (lung, colorectal, breast, prostate), number of comorbid conditions (0,1, 2, 3, 4+), time (months) from cancer diagnosis to death (0–5, 6–12, 13–36,37–60, 61–120, 121+), the status of having palliative care needs recognised (PC group) or not (non-PC group) following the cancer diagnosis. Comorbid conditions were the 17 conditions included in a modified Charlson Comorbidity Index proposed by Khan et al. [18]. The time period of counting the comorbid conditions was between the diagnosis of the concerned cancer and the death of the patient. A patient who was either on the palliative care register or had a referral record of palliative care after their cancer diagnosis was categorised as having palliative care needs recognised. The Quality and Outcomes Framework (QOF) was first introduced as part of the new General Medical Services contract in 2004 [19]. The QOF incentivises general practices to identify and register patients with palliative care needs, regularly review, assess their needs and preferences and proactively planning care. Palliative care was endorsed as a new clinical area for improvement from 2006. The needs of palliative care were identified by the recommended Read codes for palliative care QOF (See Additional file 1 QOF_codes.txt, the download link). A similar approach was successfully employed in a previous CPRD-based study which identified inequity in recognition of palliative care needs for people with heart failure [20]. The palliative care referral was identified using the National Health Service (NHS) specialty field. This contains detailed information about the referring speciality but its completion by general practice staff is not compulsory.

The socio-economic status, measured by the quintile of the index of multiple deprivation (IMD2010) (1 = least deprived to 5 = most deprived) score of the area where the practice located, was also available as an extra explanatory variable to the patient data from practices in England. The IMD score is an UK government’s official measure [21]. It is a composite score derived from seven domains: income, employment, health and disability, education skills and training, barriers to housing and services, crime and disorder, and living environment. The linkage was done by the CPRD through the postcodes of the practices with which patients were registered.

### Statistical analysis

Data were described using count and percentage for categorical variables and mean (standard deviation, SD) for continuous data where applicable. Temporal patterns of service use (consultation, prescribing and referral) in the last 12 months of life were explored using the line chart by plotting month to death against the following statistics: 1) the proportion of patients using a specific type of service with a pre-defined intensity; 2) mean number of a specific type of service. The service use pattern was explored by the status of having palliative care needs recognised or not.

To facilitate interpretations of the findings and the comparability with other studies, we categorised all continuous variables. The generalised estimating equation (GEE) was used to account for the clustering effect within the practice, meaning that the patients from the same practice tended to have similar server use patterns. Three GEE regression models were constructed to evaluate variables independently associated with the outcomes. The GEE model was built with log link function, Poisson distribution and an exchangeable working correlation matrix.

The candidate explanatory variables were selected using a combination of prior clinical knowledge and statistical criteria. The important demographical variables (e.g. age, gender) and clinical variables (e.g. cancer site, number of comorbid conditions) were forced to stay in the model regardless their statistical significance. The multiple adjusted risk ratios (aRRs) were derived from the constructed multiple regression models to quantify the association strength between the explanatory and outcome variables. Two-way interactions of explanatory variables were explored.

A similar multiple regression modelling framework was applied to identify the patient demographic and clinical characteristics associated with if a patient having palliative care needs recognosied or not.

We conducted four sensitivity analyses: 1) the service use patterns where outcome variables (number of consultations, number of prescriptions and number of referrals) were derived from the services used in the last 3 months of life; 2) using the data from practices in England only, it allowed to include IMD2010 as an extra explanatory variable; 3) using the post-2006 data only, as GP practices were incentivised from April 2006 to register patients with palliative care needs; 4) using all GP consultations involving direct patient contact only.

All analyses were performed with the Statistical Analysis Software, version 9.4 (SAS Institute, Cary, NC, USA). A two-sided *p* value of 0.05 was considered statistically significant.

## Results

### Characteristics of the study sample

Sixty-eight thousand seven hundred thirty-five patients meeting the inclusion criteria were extracted from the CPRD database. After exclusion of 212 patients with ambiguous date of diagnostic information, the final study sample comprised of 68,523 patients. The characteristics of the study sample are shown in Table 1. 70.8% of the patients were aged 70 years or above and 75.1% had one or more comorbid conditions. Nearly half of the patients (45.5%) were identified as having palliative care (PC) needs. Patients in PC group were younger, with more lung cancer and slightly more comorbidity than those in non-PC group. The median time from diagnosis to death (15 months) were similar between two groups.

**Table 1:** Characteristics* of the study population by the status of palliative care service use

Variable	Value	All	No PC	PC
N (row%)	–	68,523 (100.0)	37,330 (54.5)	31,193 (45.5)
Age at death	Median (min, max)	77 (6, 111)	78 (6110)	74 (18, 111)
	< 50	2010 (2.9)	873 (2.3)	1137 (3.6)
	50–59	5287 (7.7)	2253 (6.0)	3034 (9.7)
	60–69	12,702(18.5)	5906 (15.8)	6796 (21.8)
	70–79	21,282 (31.1)	11,258 (30.2)	10,024 (32.1)
	80–89	21,206(30.9)	12,844 (34.4)	8362 (26.8)
	90+	6036 (8.8)	4196 (11.2)	1840 (5.9)
Gender	Female	31,138(45.4)	16,805 (45.0)	14,333 (45.9)
	Male	37,385 (54.6)	20,525 (55.0)	16,860 (54.1)
Cancer site	Lung	25,154 (36.7)	11,983 (32.1)	13,171 (42.2)
	Colorectal	16,560 (24.2)	8740 (23.4)	7820 (25.1)
	Breast	13,682 (20.0)	8254 (22.1)	5428 (17.4)
	Prostate	13,127 (19.2)	8353 (22.4)	4774 (15.3)
No. of comorbid conditions	0	17,028 (24.9)	9486 (25.4)	7542 (24.2)
	1	22,774 (33.2)	12,094 (32.4)	10,680 (34.2)
	2	15,338 (22.4)	8286 (22.2)	7052 (22.6)
	3	7903 (11.5)	4335 (11.6)	3568 (11.4)
	4+	5480 (8.0)	3129 (8.4)	2351 (7.5)
Time between diagnosis and death (months)	Median (min, max)	15 (0, 292)	15 (0, 292)	15 (0, 273)
	< 6	20,172 (29.4)	11,809 (31.6)	8363 (26.8)
	6–12	11,245 (16.4)	5334 (14.3)	5911 (18.9)
	13–36	17,679 (25.8)	8674 (23.2)	9005 (28.9)
	37–60	7802 (11.4)	4283 (11.5)	3519 (11.3)
	61–120	8657 (12.6)	5279 (14.1)	3378 (10.8)
	121+	2968 (4.3)	1951 (5.2)	1017 (3.3)
Year of death	2000–2004	16,884 (24.6)	12,264 (32.9)	4620 (14.8)
	2005–2009	26,892 (39.2)	14,346 (38.4)	12,546 (40.2)
	2010–2014	24,747 (36.1)	10,720 (28.7)	14,027 (45.0)
IMD quintile**	1 (Least deprived)	8956 (13.1)	4678 (12.5)	4278 (13.7)
	2	10,019 (14.6)	5295 (14.2)	4724 (15.1)
	3	8766 (12.8)	4763 (12.8)	4003 (12.8)
	4	8076 (11.8)	4422 (11.8)	3654 (11.7)
	5 (Most deprived)	6953 (10.1)	3843 (10.3)	3110 (10.0)
	Not available	25,753 (37.6)	14,329 (38.4)	11,424 (36.6)
Region	England	53,377 (77.9)	29,214 (78.3)	24,163 (77.5)
	Wales	5921 (8.6)	3285 (8.8)	2636 (8.5)
	Scotland	7044 (10.3)	3668 (9.8)	3376 (10.8)
	Northern Ireland	2181 (3.2)	1163 (3.1)	1018 (3.3)

* expressed as N (column %) unless stated otherwise. The comparisons of the two groups were all statistically significant (*P* < 0.05)

** for England only

### Patterns of GP service use

**Fig. 1:**
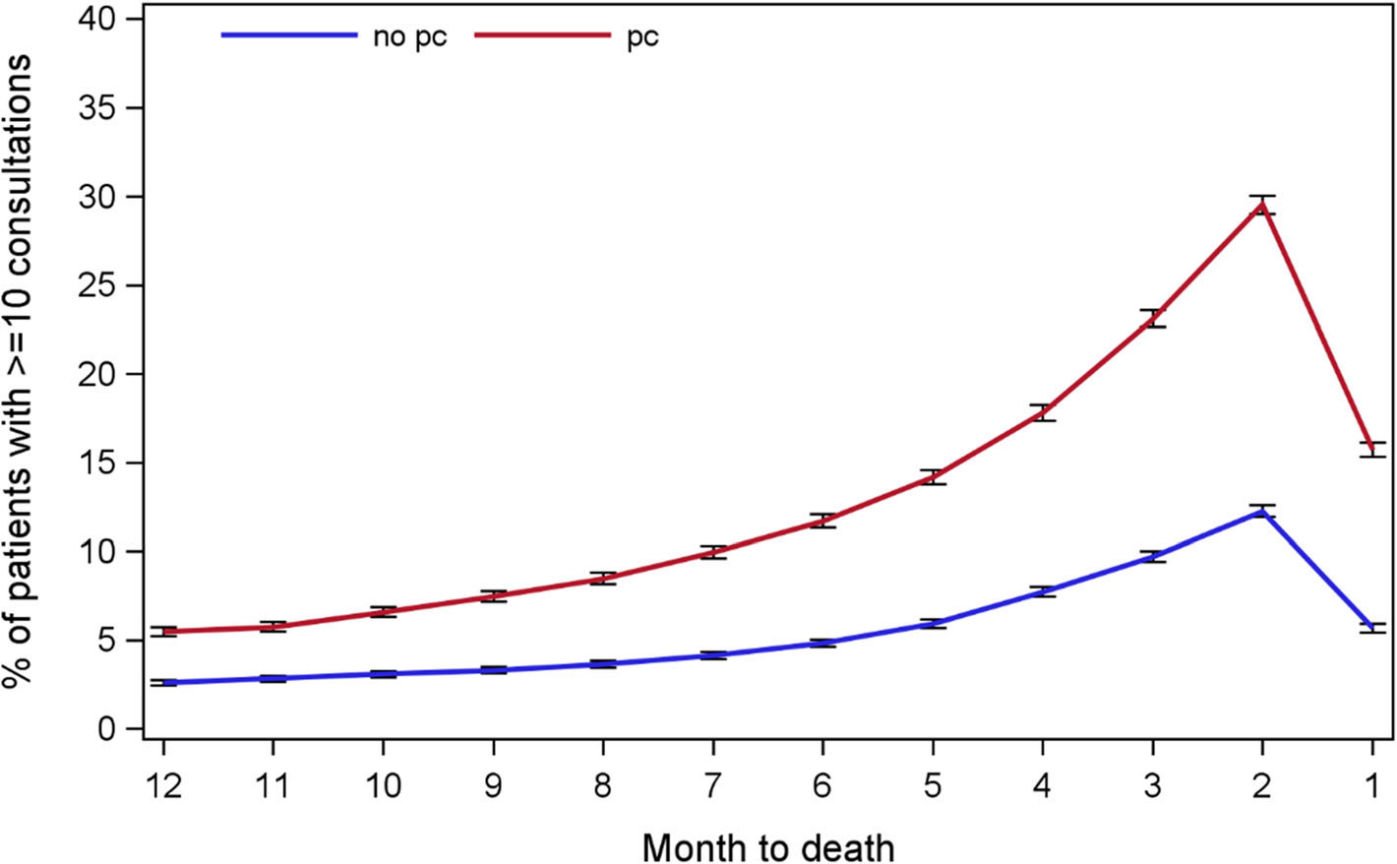
The pattern of GP consultations in the last year of life

Of the 5,819,161 consultations happening in the last year of life, 3,031,734 (52.1%) were included in this analysis. We excluded those consultations that were primarily for administrative purpose (i.e. having no direct patient contacts or those unknown types of consultations). A patient on average received 43.0 consultations in the last year of life (Standard deviation (SD): 31.7). Consultations in the last year rarely took place at the usual residences of patients (4.4%). Up to one third (29.5%) of the patients had 10 or more monthly consultations in the final year, and 15.7% in the final month. Patients in PC group received more consultations than those in non-PC group (53.3, SD: 34.4 versus 34.9, SD: 26.3) and this remained true throughout the last 12 months of life (Fig. 1). In both groups, the proportion of patients receiving 10+ consultations peaked at the last 2nd month with a sharp drop in the month before death.

**Fig. 2:**
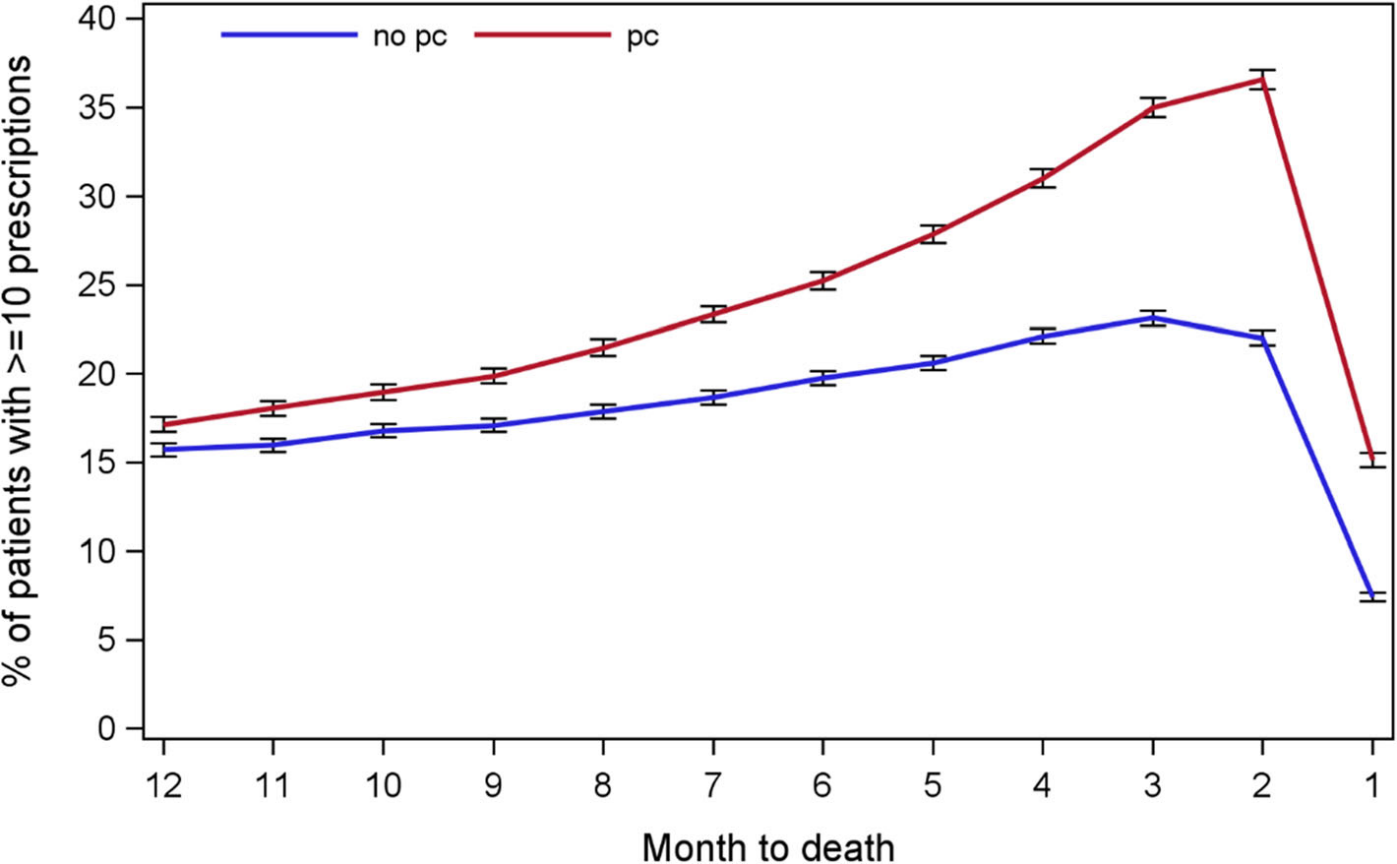
The pattern of GP prescribing in the last year of life

95.8% of the 68,523 patients in total had 5,074,178 valid GP prescribing records in the last year of life. These patients were exposed to 1313 drug groups in the year before death. A patient received a total of 71.5 prescriptions (SD: 68.0) and 21.3 different drugs (SD: 13.0) in the final year. The top five commonly prescribed drug groups were opioid analgesics (6.4%), proton pump inhibitors (4.5%), Non-opioid and compound analgesics (4.0%), antiplatelet drugs (3.9%) and Statins (3.1%). In the last 12 months, the proportion of patients receiving 10 or more monthly prescriptions from their GPs reached a peak (36.6%) in the penultimate month before death, then fell to the lowest level (15.4%) in the last month. Over the last 12 months, the GP prescribing rate was consistently higher in the PC group than in non-PC group (Fig. 2).

**Fig. 3:**
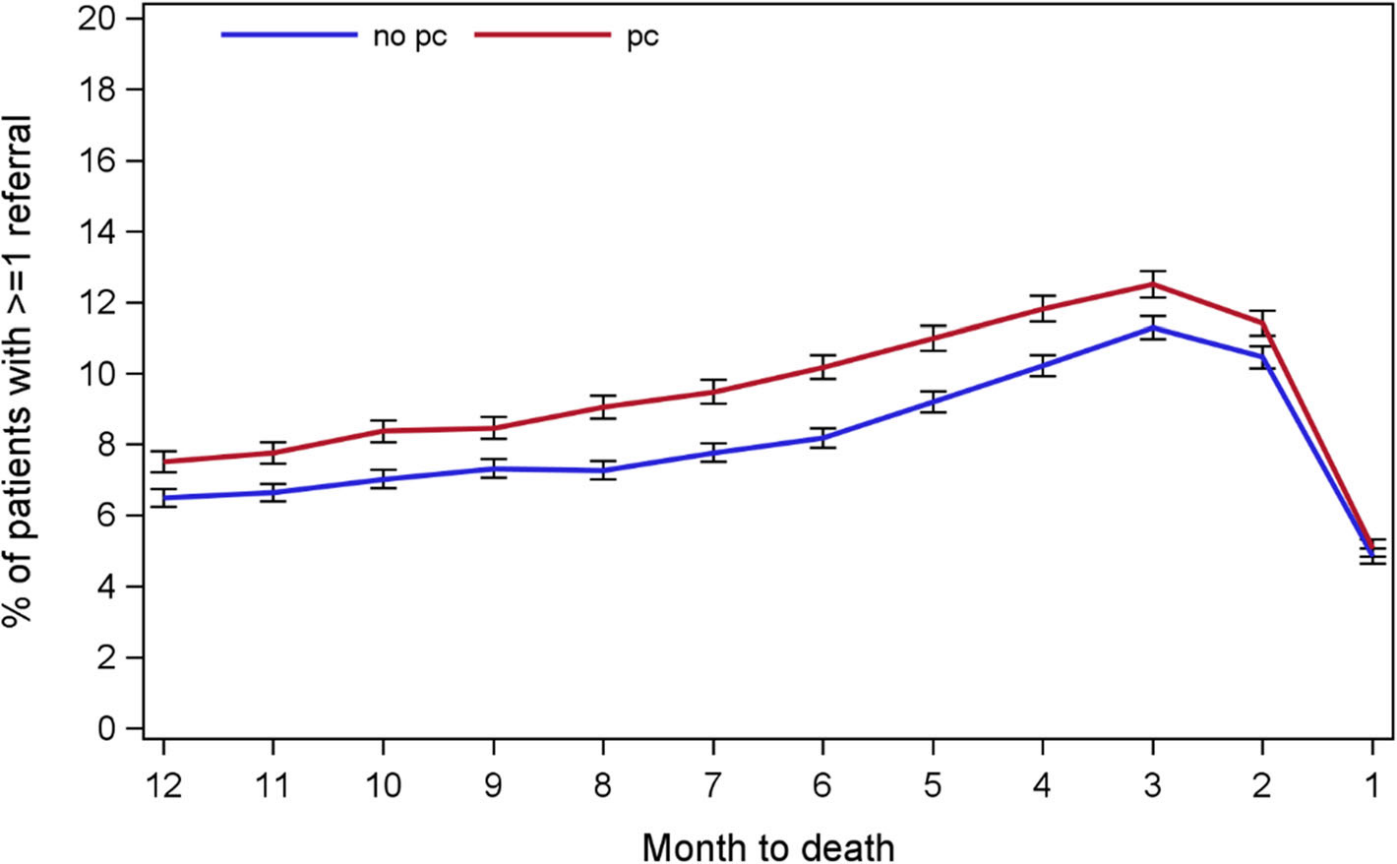
The pattern of GP referrals in the last year of life

58.0% of the study participants had at least one referral in the last year (total *N* = 89,975). The referral rate showed a slight increasing trend along with the closeness to death but remained at a lower level (7.0 to 11.8%) and dropped to the lowest (5.0%) in the month before death (Fig. 3). The average number of referral specialities fluctuated in a small range (1.2 to 1.3). Patients in PC group had a slightly higher percentage with at least one end-of-life referral record and a higher average number of referring specialities than those in the non-PC group. Even with more than one-third of missing data on referring speciaties, patients were referred to a broad range of specialities, covering two thirds (60 to 67) of the available specialties. The commonly referred specialties were general surgery or general medicine most of the time in the last year of life, and only in the last month palliative medicine became the second most referred specialty.

### Factors associated with GP service use and PC status

Patients in PC group had more GP consultations (Table 2) (aRR 1.30, 1.29–1.32), prescriptions (aRR 1.17, 1.16–1.19) and more likely to be referred for other care services (aRR 1.22, 1.19–1.26) than those in non-PC group. Increasing age was associated with fewer GP consultations but more prescriptions and a higher chance of GP referral. The effect of age on consultation started from 50 to 59 age group (aRR 0.96, 0.93 to 0.99) to the strongest in 90+ group (aRR 0.77, 0.75 to 0.80). Compared to those under 50 years old, patients aged over 50 received more prescriptions with a clear dose-response relationship (aRRs 1.09 to 1.44); while those aged between 50 and 89 were more likely to be referred to other care services (aRRs 1.08 to 1.16). Comorbidity was positively associated with consultations, prescriptions and referrals (aRRs 1.07 to 2.03), all with a dose-response relationship (p for trend < 0.0001).

**Table 2:** Factors associated with primary care service use in the 12 months of life, *N* = 68,523

Variable	Value	GP consultation		GP prescription		GP referral to other specialities		Palliative care needs recognised or not	
Palliative care	Yes	1.30(1.29 to 1.32)	< 0.0001	1.17(1.16 to 1.19)	< 0.0001	1.22(1.19 to 1.26)	< 0.0001	–	
Age at death (ref: < 50)	50–59	0.96(0.93 to 0.99)	< 0.0001	1.09(1.03 to 1.14)	< 0.0001	1.08(1.00 to 1.18)	< 0.0001	0.99(0.95 to 1.03)	< 0.0001
	60–69	0.94(0.91 to 0.96)		1.18(1.12 to 1.24)		1.13(1.04 to 1.23)		0.89(0.86 to 0.93)	
	70–79	0.92(0.89 to 0.94)		1.24(1.18 to 1.30)		1.16(1.07 to 1.25)		0.81(0.78 to 0.84)	
	80–89	0.85(0.83 to 0.88)		1.32(1.25 to 1.39)		1.13(1.04 to 1.22)		0.69(0.66 to 0.72)	
	90+	0.77(0.75 to 0.80)		1.44(1.36 to 1.52)		0.99(0.91 to 1.09)		0.53(0.50 to 0.56)	
Gender (ref: female)	Male	0.96(0.95 to 0.97)	< 0.0001	0.90(0.89 to 0.92)	< 0.0001	0.99(0.97 to 1.02)	0.51	0.97(0.95 to 0.99)	0.003
Cancer site (ref: Lung)	Colorectal	0.98(0.97 to 1.00)	< 0.0001	1.05(1.03 to 1.08)	< 0.0001	1.04(1.01 to 1.09)	< 0.0001	0.78(0.75 to 0.80)	< 0.0001
	Breast	1.01(1.00 to 1.02)		0.96(0.95 to 0.98)		1.00(0.97 to 1.03)		0.91(0.89 to 0.93)	
	Prostate	1.19(1.17 to 1.21)		1.14(1.12 to 1.17)		1.21(1.17 to 1.26)		0.76(0.74 to 0.79)	
No. comorbid conditions (ref: 0)	1	1.09(1.08 to 1.11)	< 0.0001	1.21(1.18 to 1.23)	< 0.0001	1.07(1.03 to 1.11)	< 0.0001	1.07(1.04 to 1.09)	< 0.0001
	2	1.20(1.19 to 1.22)		1.45(1.42 to 1.48)		1.14(1.10 to 1.18)		1.08(1.05 to 1.10)	
	3	1.17(1.15 to 1.19)		1.68(1.64 to 1.72)		1.24(1.19 to 1.29)		1.08(1.04 to 1.11)	
	4+	1.26(1.24 to 1.28)		2.03(1.98 to 2.09)		1.32(1.25 to 1.39)		1.01(0.97 to 1.05)	
Time between diagnosis and death (ref: 120+)	0–5	1.07(1.04 to 1.11)	0.0001	0.95(0.91 to 0.99)	< 0.0001	1.53(1.42 to 1.65)	< 0.0001	1.11(1.06 to 1.17)	< 0.0001
	6–12	1.31(1.27 to 1.35)		1.07(1.02 to 1.12)		1.41(1.30 to 1.52)		1.38(1.31 to 1.45)	
	13–36	1.10(1.07 to 1.13)		1.05(1.01 to 1.09)		0.92(0.86 to 0.99)		1.41(1.34 to 1.49)	
	37–60	1.05(1.01 to 1.08)		1.03(0.98 to 1.07)		0.96(0.89 to 1.05)		1.32(1.25 to 1.39)	
	61–120	1.01(0.98 to 1.03)		0.98(0.94 to 1.02)		0.98(0.91 to 1.05)		1.17(1.11 to 1.23)	
Year of death (ref: 2010–2014)	2000–2004	0.64(0.62 to 0.66)	< 0.0001	0.79(0.77 to 0.80)	< 0.0001	1.13(1.01 to 1.25)	< 0.0001	0.48(0.45 to 0.51)	< 0.0001
	2005–2009	0.86(0.84 to 0.88)		0.91(0.89 to 0.93)		0.93(0.88 to 0.97)		0.81(0.78 to 0.83)	
Region (ref: England)	Wales	0.85(0.75 to 0.96)	0.005	1.15(1.08 to 1.22)	< 0.0001	1.23(1.01 to 1.49)	0.007	0.98(0.91 to 1.06)	0.62
	Scotland	1.06(0.96 to 1.17)		0.87(0.83 to 0.91)		0.77(0.61 to 0.97)		0.99(0.93 to 1.05)	
	Northern Ireland	1.34(1.09 to 1.64)		1.01(0.93 to 1.09)		1.14(0.95 to 1.35)		0.91(0.78 to 1.06)	

Older age (≥60 years) was associated with a lower chance of having palliative care needs recognised. There was a dose-response relationship between age at death and the chance of PC group membership – aRRs ranging from 0.53 in 90+ to 0.89 in 60–69 age group. Patients with comorbidities were more likely to be in the PC group (aRRs 1.07 to 1.08).

Sensitivity analyses (Appendix Tables 3–6) demonstrated similar results.

## Discussion

### Main findings and implications

In this large and nationally representative, retrospective cohort study, we found that most cancer patients managed by GPs have complex care needs [22], i.e. ≥70 years, with comorbidities. GPs maintain active contacts with terminal cancer patients towards the very end of their lives, their involvement reaching a peak at the penultimate month before a sharp drop in the last month. Polypharmacy is common. Patients over 70 years have fewer GP consultations but more prescriptions and a higher chance of referral to other specialities, suggesting their care needs may not be sufficiently met by primary care teams. Having palliative care needs recognised increases the use of GP services, however, patients of old age have a significantly lower chance of use palliative care services. These data provide a valuable insight into the past and current practice in the UK primary care. These could be used to, for example, highlight areas needing attention and inform the development of clinical guidelines. These data are also useful as a baseline for subsequent studies examining primary care’s role in PEoLC, both within and beyond the UK.

Over the next several decades, it is anticipated that primary care teams will experience more pressure to deliver care to those who are dying, as the patients with complex care needs will continue to rise and increasingly more people will die in the community settings [23]. Our data together with these projected trends and people’s preferences to be cared for in the community [3] highlight the importance of enhancing provision of primary palliative care. As most cancer patients have one or more comorbidities, our data also challenge the single-disease oriented primary care system, particularly the appropriateness of the widely implemented time-limited consultation model and ‘one problem per consultation’ policy in some of the UK practices.

The skill sets for primary palliative care include basic symptom management and psychological support, basic discussions about prognosis, treatment goals and advance care planning [24]. A previous study involving 513 GPs found that more than a quarter of the respondents reported receiving no or inadequate training in the delivery of end of life care [25]. A Danish study on GPs’ self-reported competences in end of life care (*n* = 573) found a similar proportion of the respondents don’t feel confident about being a key care provider [26]. Another qualitative study concerning GPs’ educational needs also reported a widespread lack of confidence in end of life care amongst both qualified and trainee GPs [27]. Our data support these findings and prompt actions to address the gaps in primary care providers.

A finding worth noting is the diverse needs of care among terminal cancer patients, as evident by a broad range of referral specialities throughout the last year of life. The primary care teams maintain active contacts with patients towards the very end of their lives, which offers opportunities for a greater integration of primary care with specialist palliative care, oncology, geriatrics and other healthcare specialities. In fact, the primary plus specialist palliative care has been proposed as a sustainable model to meet the high demand for palliative care [24]. Furthermore, GPs are well positioned and should play a key role to coordinate patients’ care among multiple healthcare teams [28].

Polypharmacy is common in our study population. In the 2nd to last month of life GPs were still actively prescribing for patients, as high as 36% of the patient received 10 or more prescriptions from their GPs. A Swedish study using linked data covering all settings reported a slightly higher number (10+) of medications [29] than our figures, particularly towards end of life. It may be primarily due to the CPRD data only including the medications prescribed by the GPs. Polypharmacy around the end of life may also involve inappropriate prescribing, a previous study estimated that this proportion may be as high as one third among centanarians [30]. Polypharmacy has been shown to increase the symptom burden, the risk of adverse drug effects and even mortality [31], future research should be directed to safe and effective deprescribing for end of life care patients. Tools like the Drug Burden Index [32], which measures cumulative exposure to anticholinergic and sedative drugs or other measures of anticholinergic burden, may help guide drug choices at the end of life.

Although age-related inequalities in end of life care are reported extensively in the literature [33, 34], this study provides to date the largest population-based evidence from the primary care setting. Eradicating inequalities in access to care and providing person-centered, coordinated high quality end of life care to everyone is a central commitment of our health and social care systems [35]. A recent study (*N* = 2479) using the local cancer registry database linked to GP and hospital activity data found that access to and longer duration with palliative care were associated with better end-of-life quality indicators [36]. Our data showed that palliative care was associated with the increased use of primary care services, but older people were less likely to utilise palliative care services, which may be due to their generally having less clear early triggers for palliative care [37] or other potential confounders (e.g. impairments) [38]. This needs to be investigated further in future studies to inform the design of effective interventions to improve the situation.

### Strengths and limitations

This is the largest evaluation study of primary care service use at the end of life in cancer patients. Due to the strength of the database, we were able to track all the interactions between patients and primary care in the last year of life. We also, for the first time, found that end-of-life cancer patients tend to use GP services more if their palliative care needs are known to their GPs. However, we could not assess the service utilisation in the context of non-GP health care activities due to the restrictions of record linkage at the time of our data access application. The capacity of the CPRD linking to other data sources has been greatly enhanced in recent years. The primary care database can now be linked to the whole spectrum of hospital episode statistics, cancer diagnosis and treatment data, and mental health data. Future studies should maximise the opportunities to acquire linked datasets to gain a more comprehensive picture of services patients received during the end of life, for example, the role of GPs in the continuity of end of life care and how it is related to care outcomes, how GPs can extend their collaboaration with cancer services. Furthermore, as the CPRD prescription data only indicate whether a prescription has been issued and not whether it was dispensed or taken as recommended. The CPRD prescribing data also do not capture the medications prescribed outside GP care settings or over-the-counter medication use [16]. Finally, we do not have information on the family or caregivers who could influence the GP service use (e.g. number of consultations, prescriptions). We also do not have information on preferences, which could influence the referrals to palliative care.

## Conclusions

We found most terminal cancer patients managed by GPs have complex care needs, i.e. ≥70 years, three quarters with comorbidities. GPs maintain active contacts with terminal cancer patients towards the very end of their lives. Polypharmacy is common and referrals happen even in the last month of life. Patients aged over 70 years have fewer GP consultations but more prescriptions and a higher chance of end of life referral to other specialities. Having palliative care needs recognised increases the use of GP services, however, patients of old age have a significantly lower chance of being in the PC group. Our data highlight the importance of enhancing primary palliative care provision and the needs for a greater integration of primary care, and other healthcare professionals. Age-related inequalities in EoL care - older patients have poorer access to both primary care and palliative care - need to be addressed in future studies.

### Supplementary information

**Additional file 1.**

## Data Availability

According to the data agreement we signed with the data provider, we are not allowed to share our data. Access to the data would need special approvals from the CPRD office and is subject to protocol approval by an Independent Scientific Advisory Committee (ISAC). More details can be found on the data access section of the CPRD website. This study was approved by the ISAC for Medicines and Healthcare Products Regulatory Agency (MHRA) database research permission (Approval number: 09_035RMn).

## Appendix

**Table 3:** Factors associated with primary care service use in the last 3 months of life, *N* = 68,523

Variable	Value	GP consultation		GP prescription		GP referral to other specialities	
Palliative care	Yes	1.39(1.37 to 1.42)	< 0.0001	1.39(1.37 to 1.41)	< 0.0001	1.26(1.20 to 1.33)	< 0.0001
Age at death (ref: < 50)	50–59	0.99(0.95 to 1.02)	< 0.0001	1.04(0.98 to 1.10)	< 0.0001	1.14(1.00 to 1.30)	< 0.0001
	60–69	0.97(0.94 to 1.01)		1.05(0.99 to 1.10)		1.24(1.09 to 1.41)	
	70–79	0.93(0.90 to 0.96)		1.03(0.97 to 1.08)		1.23(1.09 to 1.40)	
	80–89	0.86(0.83 to 0.90)		1.04(0.98 to 1.10)		1.23(1.08 to 1.39)	
	90+	0.81(0.78 to 0.84)		1.12(1.06 to 1.20)		1.12(0.97 to 1.28)	
Gender (ref: female)	Male	0.97(0.96 to 0.98)	< 0.0001	0.90(0.89 to 0.92)	< 0.0001	1.04(1.00 to 1.08)	0.05
Cancer site (ref: Lung)	Colorectal	0.99(0.97 to 1.01)	< 0.0001	1.01(0.98 to 1.04)	< 0.0001	1.10(1.04 to 1.16)	< 0.0001
	Breast	1.00(0.98 to 1.02)		0.95(0.93 to 0.98)		0.99(0.95 to 1.04)	
	Prostate	1.10(1.08 to 1.12)		1.11(1.08 to 1.13)		1.15(1.09 to 1.22)	
No. comorbidity conditions (ref: 0)	1	1.03(1.01 to 1.04)	< 0.0001	1.11(1.09 to 1.14)	< 0.0001	1.02(0.96 to 1.08)	< 0.0001
	2	1.08(1.06 to 1.10)		1.26(1.23 to 1.28)		1.05(0.99 to 1.11)	
	3	1.10(1.08 to 1.12)		1.39(1.35 to 1.43)		1.09(1.02 to 1.16)	
	4+	1.18(1.16 to 1.20)		1.58(1.53 to 1.63)		1.17(1.07 to 1.27)	
Time between diagnosis and death (months) (ref: 120+)	< 6	1.40(1.35 to 1.45)	< 0.0001	1.15(1.10 to 1.21)	< 0.0001	1.81(1.62 to 2.02)	< 0.0001
	6–12	1.14(1.10 to 1.19)		1.12(1.07 to 1.18)		1.04(0.93 to 1.17)	
	13–36	1.09(1.05 to 1.13)		1.07(1.03 to 1.12)		1.01(0.91 to 1.13)	
	37–60	1.06(1.02 to 1.10)		1.05(1.00 to 1.10)		1.03(0.91 to 1.16)	
	61–120	1.00(0.96 to 1.04)		1.00(0.95 to 1.04)		1.06(0.95 to 1.18)	
Year of death (ref: 2010–2014)	2000–2004	0.66(0.63 to 0.69)	< 0.0001	0.88(0.85 to 0.90)	< 0.0001	1.16(1.03 to 1.31)	< 0.0001
	2005–2009	0.85(0.83 to 0.88)		0.93(0.91 to 0.96)		0.92(0.86 to 0.99)	
Region (ref: England)	Wales	0.91(0.81 to 1.02)	0.12	1.10(1.03 to 1.17)	< 0.0001	1.23(1.00 to 1.51)	0.029
	Scotland	0.99(0.88 to 1.10)		0.86(0.81 to 0.92)		0.83(0.64 to 1.07)	
	NI	1.22(1.00 to 1.48)		1.07(0.95 to 1.20)		1.21(1.01 to 1.46)	

**Table 4:** Factors associated with primary care service use in the last 12 months of life, sensitivity analysis with IMD, *N* = 42,770

		GP consultation		GP prescription		GP referral to other specialities		Palliative care needs recognised or not	
Palliative care	Yes	1.29(1.27 to 1.31)	< 0.0001	1.16(1.14 to 1.18)	< 0.0001	1.21(1.17 to 1.25)	< 0.0001		
Age at death (ref: < 50)	50–59	0.96(0.93 to 1.00)	< 0.0001	1.08(1.00 to 1.15)	< 0.0001	1.10(0.99 to 1.22)	< 0.0001	0.98(0.93 to 1.03)	< 0.0001
	60–69	0.94(0.91 to 0.97)		1.20(1.13 to 1.28)		1.15(1.04 to 1.27)		0.88(0.84 to 0.93)	
	70–79	0.92(0.90 to 0.95)		1.28(1.20 to 1.36)		1.16(1.05 to 1.27)		0.80(0.76 to 0.84)	
	80–89	0.86(0.83 to 0.89)		1.37(1.28 to 1.46)		1.14(1.03 to 1.25)		0.67(0.64 to 0.71)	
	90+	0.79(0.76 to 0.82)		1.50(1.39 to 1.61)		0.99(0.88 to 1.10)		0.53(0.49 to 0.56)	
Gender (ref: female)	Male	0.96(0.95 to 0.97)	< 0.0001	0.91(0.89 to 0.92)	< 0.0001	0.98(0.95 to 1.01)	0.14	0.97(0.95 to 1.00)	0.033
Cancer site (ref: Lung)	Colorectal	0.99(0.97 to 1.01)	< 0.0001	1.07(1.04 to 1.10)	< 0.0001	1.05(1.00 to 1.10)	< 0.0001	0.76(0.73 to 0.79)	< 0.0001
	Breast	1.01(1.00 to 1.02)		0.97(0.94 to 0.99)		1.02(0.98 to 1.06)		0.90(0.87 to 0.92)	
	Prostate	1.20(1.18 to 1.22)		1.16(1.13 to 1.19)		1.24(1.18 to 1.30)		0.76(0.73 to 0.80)	
No. comorbid conditions (ref: 0)	1	1.09(1.07 to 1.10)	< 0.0001	1.19(1.17 to 1.22)	< 0.0001	1.07(1.03 to 1.12)	< 0.0001	1.06(1.03 to 1.09)	< 0.0001
	2	1.20(1.18 to 1.22)		1.45(1.41 to 1.49)		1.15(1.10 to 1.21)		1.07(1.04 to 1.11)	
	3	1.17(1.14 to 1.19)		1.66(1.61 to 1.72)		1.24(1.18 to 1.31)		1.08(1.03 to 1.12)	
	4+	1.25(1.23 to 1.28)		2.07(2.00 to 2.15)		1.38(1.29 to 1.48)		1.00(0.95 to 1.06)	
Time between diagnosis and death (ref: 120+)	0–5	1.08(1.04 to 1.11)	< 0.0001	0.96(0.91 to 1.01)	< 0.0001	1.56(1.43 to 1.71)	< 0.0001	1.09(1.03 to 1.16)	< 0.0001
	6–12	1.31(1.26 to 1.35)		1.07(1.01 to 1.13)		1.47(1.34 to 1.61)		1.37(1.29 to 1.46)	
	13–36	1.09(1.06 to 1.13)		1.04(1.00 to 1.10)		0.95(0.87 to 1.04)		1.39(1.31 to 1.48)	
	37–60	1.05(1.01 to 1.08)		1.03(0.98 to 1.09)		1.00(0.91 to 1.10)		1.29(1.21 to 1.37)	
	61–120	1.01(0.97 to 1.04)		0.97(0.92 to 1.02)		1.02(0.93 to 1.11)		1.16(1.09 to 1.24)	
Year of death (ref: 2010–2014)	2000–2004	0.64(0.61 to 0.67)	< 0.0001	0.77(0.75 to 0.80)	< 0.0001	1.07(0.94 to 1.22)	< 0.0001	0.50(0.47 to 0.54)	< 0.0001
	2005–2009	0.85(0.82 to 0.87)		0.90(0.87 to 0.92)		0.89(0.84 to 0.94)		0.84(0.81 to 0.87)	
IMD (ref: most deprived)	Least depr	1.02(0.99 to 1.04)	0.24	0.86(0.83 to 0.89)	< 0.0001	1.07(0.99 to 1.14)	0.10	1.09(1.04 to 1.13)	< 0.0001
	2	1.02(1.00 to 1.04)		0.89(0.87 to 0.92)		1.06(0.99 to 1.13)		1.09(1.05 to 1.13)	
	3	1.02(0.99 to 1.04)		0.91(0.88 to 0.94)		1.03(0.96 to 1.10)		1.03(0.99 to 1.07)	
	4	1.00(0.98 to 1.02)		0.93(0.90 to 0.96)		1.01(0.95 to 1.07)		1.01(0.98 to 1.05)	

**Table 5:** Factors associated with primary care service use in the last 12 months of life, using all deaths post QoF, *N* = 46,703

		GP consultation		GP prescription		GP referral to other specialities		Palliative care needs recognised or not	
Palliative care (ref: Yes)	No	1.32(1.31 to 1.34)	< 0.0001	1.17(1.15 to 1.19)	< 0.0001	1.23(1.19 to 1.26)	< 0.0001		
Age at death (ref: < 50)	50–59	0.95(0.92 to 0.99)	< 0.0001	1.13(1.06 to 1.20)	< 0.0001	1.10(1.00 to 1.20)	< 0.0001	0.98(0.94 to 1.02)	< 0.0001
	60–69	0.93(0.90 to 0.97)		1.19(1.12 to 1.26)		1.18(1.08 to 1.28)		0.88(0.85 to 0.92)	
	70–79	0.90(0.87 to 0.94)		1.27(1.20 to 1.34)		1.25(1.15 to 1.35)		0.81(0.78 to 0.85)	
	80–89	0.84(0.81 to 0.87)		1.36(1.29 to 1.44)		1.23(1.13 to 1.34)		0.70(0.67 to 0.73)	
	90+	0.78(0.75 to 0.81)		1.49(1.40 to 1.59)		1.10(0.99 to 1.21)		0.56(0.53 to 0.59)	
Gender (ref: female)	Male	0.95(0.94 to 0.96)	< 0.0001	0.90(0.88 to 0.91)	< 0.0001	1.00(0.97 to 1.03)	0.96	0.97(0.96 to 0.99)	0.006
Cancer site (ref: Lung)	Colorectal	1.00(0.98 to 1.02)	< 0.0001	1.06(1.03 to 1.09)	< 0.0001	1.06(1.01 to 1.10)	< 0.0001	0.78(0.75 to 0.80)	< 0.0001
	Breast	1.03(1.02 to 1.04)		0.97(0.95 to 0.99)		0.99(0.95 to 1.02)		0.90(0.88 to 0.93)	
	Prostate	1.21(1.19 to 1.23)		1.14(1.11 to 1.17)		1.19(1.14 to 1.24)		0.76(0.73 to 0.78)	
No. comorbid conditions (ref: 0)	1	1.09(1.06 to 1.13)	< 0.0001	1.20(1.17 to 1.23)	< 0.0001	1.05(1.01 to 1.09)	< 0.0001	1.06(1.03 to 1.08)	< 0.0001
	2	1.33(1.29 to 1.37)		1.45(1.42 to 1.49)		1.13(1.09 to 1.17)		1.06(1.03 to 1.09)	
	3	1.11(1.07 to 1.14)		1.69(1.64 to 1.74)		1.21(1.16 to 1.26)		1.05(1.02 to 1.08)	
	4+	1.06(1.03 to 1.10)		2.04(1.97 to 2.10)		1.30(1.24 to 1.38)		0.99(0.95 to 1.03)	
Time between diagnosis and death (ref: 120+)	< 6	1.01(0.98 to 1.04)	0 < .0001	0.97(0.93 to 1.02)	< 0.0001	1.54(1.42 to 1.67)	< 0.0001	1.13(1.07 to 1.20)	< 0.0001
	6–12	0.79(0.77 to 0.81)		1.08(1.03 to 1.13)		1.43(1.32 to 1.54)		1.38(1.31 to 1.45)	
	13–36	0.89(0.81 to 0.98)		1.06(1.01 to 1.10)		0.90(0.84 to 0.98)		1.42(1.35 to 1.50)	
	37–60	1.04(0.95 to 1.14)		1.03(0.98 to 1.08)		0.93(0.86 to 1.01)		1.32(1.25 to 1.40)	
	61–120	1.30(1.09 to 1.54)		0.98(0.94 to 1.03)		0.95(0.88 to 1.02)		1.17(1.11 to 1.24)	
Year of death (ref: 2010–2014)	2006–2009	1.18(1.16 to 1.19)	< 0.0001	0.92(0.90 to 0.94)	< 0.0001	0.93(0.88 to 0.97)	0.003	0.86(0.84 to 0.88)	< 0.0001
Region (ref: England)	Wales	1.24(1.22 to 1.26)	0.011	1.14(1.07 to 1.22)	< 0.0001	1.19(1.00 to 1.42)	< 0.0001	0.98(0.91 to 1.05)	0.50
	Scotland	1.07(1.06 to 1.09)		0.85(0.80 to 0.89)		0.62(0.49 to 0.77)		1.00(0.95 to 1.06)	
	NI	1.34(1.31 to 1.37)		1.00(0.92 to 1.08)		1.35(1.10 to 1.66)		0.91(0.79 to 1.04)	

**Table 6:** Factors associated with service use in the 12 months of life, *N* = 68,523

Variable	Value	GP consultation^a^		GP prescription^b^	
Palliative care	Yes	1.30(1.29 to 1.32)	< 0.0001	1.17(1.16 to 1.18)	< 0.0001
Age at death (ref: < 50)	50–59	0.98(0.95 to 1.01)	< 0.0001	1.09(1.04 to 1.13)	< 0.0001
	60–69	0.97(0.95 to 1.00)		1.21(1.16 to 1.25)	
	70–79	0.95(0.93 to 0.98)		1.26(1.22 to 1.31)	
	80–89	0.90(0.88 to 0.93)		1.28(1.23 to 1.33)	
	90+	0.85(0.83 to 0.88)		1.34(1.29 to 1.40)	
Gender (ref: female)	Male	0.96(0.95 to 0.97)	< 0.0001	0.93(0.91 to 0.94)	< 0.0001
Cancer site (ref: Lung)	Colorectal	1.01(1.00 to 1.03)	< 0.0001	1.06(1.04 to 1.08)	< 0.0001
	Breast	1.04(1.03 to 1.05)		0.97(0.96 to 0.99)	
	Prostate	1.22(1.20 to 1.24)		1.15(1.13 to 1.17)	
No. comorbidity conditions (ref: 0)	1	1.09(1.08 to 1.11)	< 0.0001	1.20(1.18 to 1.22)	< 0.0001
	2	1.21(1.19 to 1.22)		1.43(1.40 to 1.45)	
	3	1.30(1.28 to 1.32)		1.62(1.59 to 1.65)	
	4+	1.42(1.39 to 1.44)		1.92(1.88 to 1.95)	
Time between diagnosis and death (months) (ref: 120+)	< 6	1.06(1.03 to 1.09)	< 0.0001	0.99(0.95 to 1.02)	
	6–12	1.27(1.23 to 1.31)		1.09(1.05 to 1.12)	< 0.0001
	13–36	1.09(1.06 to 1.11)		1.06(1.03 to 1.10)	
	37–60	1.04(1.01 to 1.07)		1.04(1.01 to 1.07)	
	61–120	1.00(0.97 to 1.03)		1.00(0.97 to 1.03)	
Year of death (ref: 2010–2014)	2000–2004	0.63(0.61 to 0.65)	< 0.0001	0.82(0.81 to 0.84)	< 0.0001
	2005–2009	0.87(0.85 to 0.88)		0.93(0.91 to 0.94)	
Region (ref: England)	Wales	0.98(0.92 to 1.04)	< 0.0001	1.14(1.09 to 1.19)	< 0.0001
	Scotland	1.13(1.06 to 1.20)		0.98(0.94 to 1.02)	
	NI	1.57(1.43 to 1.71)		1.15(1.07 to 1.24)	

^a^ including all consultations (i.e. direct & indirect patient contacts); ^b^prescriptions of unique products only

## Abbreviations

CPRD: The Clinical Practice Research DataLink
EoL: End of Life
GEE: The Generalised Estimation Eq.
GP: General Practice
GPs: General Practitioners
IMD: The Index of Multiple Deprivation
ISAC: The Independent Scientific Advisory Committee
MHRA: The Medicines and Healthcare Products Regulatory Agency
NHS: The National Health Service
QOF: The Quality and Outcomes Framework
PC: Palliative Care

## References

[1] Aldridge MD, Kelley AS. The Myth Regarding the High Cost of End-of-Life Care. 2015;105:2411–5. 10.2105/AJPH.2015.302889 PMC4638261 26469646

[2] Skorstengaard MH, Neergaard MA, Andreassen P, Brogaard T, Bendstrup E, Lokke A (2017). Preferred Place of Care and Death in Terminally Ill Patients with Lung and Heart Disease Compared to Cancer Patients. J Palliat Med.

[3] Gomes B, Higginson IJ, Calanzani N, Cohen J, Deliens L, Daveson BA (2012). Preferences for place of death if faced with advanced cancer: a population survey in England, Flanders, Germany, Italy, the Netherlands, Portugal and Spain. Ann Oncol.

[4] Gomes B, Calanzani N, Higginson I (2011). Local preferences and place of death in regions within England 2010.

[5] Department of Health (2008). End of Life Care Strategy: Promoting high quality care for all adults at the end of life.

[6] Institute of Medicine (2015). Dying in America: Improving Quality and Honoring Individual Preferences Near the End of Life.

[7] PACE - Resolution 2249 (2018) - The provision of palliative care in Europe. http://tiny.cc/f92klz. Accessed 19 Mar 2020.

[8] Cohen J, Pivodic L, Miccinesi G, Onwuteaka-Philipsen BD, Naylor WA, Wilson DM (2015). International study of the place of death of people with cancer: a population-level comparison of 14 countries across 4 continents using death certificate data. Br J Cancer.

[9] Lien foundation, The Economist Intelligence Unit (2015). The 2015 Quality of Death Index Ranking palliative care across the world.

[10] Office for National Statistics (2016). National Survey of Bereaved People (VOICES), 2015.

[11] Starfield B, Shi L, Macinko J, Milbank Q (2005). Contribution of primary care to health systems and health. Milbank Q.

[12] de Nooijer K, Pivodic L, Deliens L, Miccinesi G, Vega Alonso T, Moreels S, et al. Primary palliative care for older people in three European countries: a mortality follow-back quality study. BMJ Support Palliat Care. 2019. 10.1136/bmjspcare-2019-001967. 10.1136/bmjspcare-2019-001967 PMC7691801 31619438

[13] Abarshi E, Echteld MA, Van den Block L, Donker G, Bossuyt N, Meeussen K (2011). Use of Palliative Care Services and General Practitioner Visits at the End of Life in The Netherlands and Belgium. J Pain Symptom Manag.

[14] Penders YW, Onwuteaka-Philipsen B, Moreels S, Donker GA, Miccinesi G, Alonso TV (2018). Differences in primary palliative care between people with organ failure and people with cancer: An international mortality follow-back study using quality indicators. Palliat Med.

[15] Van Der Plas AGM, Oosterveld-Vlug MG, Pasman HR, Schweitzer B, Onwuteaka-Philipsen BD (2018). Continuity of GP care after the last hospitalization for patients who died from cancer, chronic obstructive pulmonary disease or heart failure: A retrospective cohort study using administrative data. Fam Pract.

[16] Herrett E, Gallagher AM, Bhaskaran K, Forbes H, Mathur R, Staa T Van, et al. Data Resource Profile: Clinical Practice Research Datalink (CPRD). 2015:827–36. 10.1093/ije/dyv098 PMC4521131 26050254

[17] NHS Digital. Read Codes.

[18] Khan NF, Perera R, Harper S, Rose PW (2010). Adaptation and validation of the Charlson Index for Read/OXMIS coded databases. BMC Fam Pract.

[19] Free A, Thomas K, Walton W, Griffin T. Full Guidance on Using QOF to Improve Palliative/End of Life Care in Primary Care. Gold Standard Framework. 2006. http://tiny.cc/613klz. Accessed 19 Mar 2020.

[20] Gadoud A, Kane E, Macleod U, Ansell P, Oliver S, Johnson M (2014). Palliative care among heart failure patients in primary care: a comparison to cancer patients using English family practice data. PLoS One.

[21] Department for Communities and Local Government (2010). The English Indices of Deprivation 2010: Statistical Release.

[22] Min L, Wenger N, Walling AM, Blaum C, Cigolle C, Ganz DA (2013). When Comorbidity, Aging, and Complexity of Primary Care Meet: Development and Validation of the Geriatric CompleXity of Care Index. J Am Geriatr Soc.

[23] Bone AE, Gomes B, Etkind SN, Verne J, Murtagh FE, Evans CJ (2018). What is the impact of population ageing on the future provision of end-of-life care? Population-based projections of place of death. Palliat Med.

[24] Quill TE, Abernethy A. Generalist plus Specialist Palliative Care — Creating a More Sustainable Mode. JAMA. 2013. 10.1056/NEJMp1215620. 10.1056/NEJMp1215620 23465068

[25] Mitchell S, Loew J, Millington-Sanders C, Dale J (2016). Providing end-of-life care in general practice: findings of a national GP questionnaire survey. Br J Gen Pract.

[26] Winthereik A, Neergaard M, Vedsted P, Jensen A (2016). Danish general practitioners’ self-reported competences in end-of-life care. Scand J Prim Health Care.

[27] Selman LE, Brighton LJ, Robinson V, George R, Khan SA, Burman R (2017). Primary care physicians’ educational needs and learning preferences in end of life care: A focus group study in the UK. BMC Palliat Care.

[28] Borgsteede SD, Graafland-Riedstra C, Deliens L, Francke AL, van Eijk JTM, Willems DL (2006). Good end-of-life care according to patients and their GPs. Br J Gen Pract.

[29] Morin L, Vetrano DL, Rizzuto D, Calderón-Larrañaga A, Fastbom J, Johnell K (2017). Choosing Wisely? Measuring the Burden of Medications in Older Adults near the End of Life: Nationwide, Longitudinal Cohort Study. Am J Med.

[30] Hazra NC, Dregan A, Jackson S, Gulliford MC (2016). Drug Utilization and Inappropriate Prescribing in Centenarians. J Am Geriatr Soc.

[31] Schenker Y, Park SY, Jeong K, Pruskowski J, Kavalieratos D, Resick J, et al. Associations Between Polypharmacy, Symptom Burden, and Quality of Life in Patients with Advanced, Life-Limiting Illness. J Gen Intern Med. 2019. 10.1007/s11606-019-04837-7. 10.1007/s11606-019-04837-7 PMC6445911 30719645

[32] Hilmer SN, Mager DE, Simonsick EM, Cao Y, Ling SM, Windham BG (2007). A Drug Burden Index to Define the Functional Burden of Medications in Older People. Arch Intern Med.

[33] CQC. A different end: Addressing inequalities in end of life care. 2016;41. http://tiny.cc/hb5klz. Accessed 19 Mar 2020.

[34] Gao W, Ho YK, Verne J, Glickman M, Higginson IJ, GUIDE_Care project (2013). Changing Patterns in Place of Cancer Death in England: A Population-Based Study. PLoS Med.

[35] Partnership NP, E of LC (2016). Ambitions for Palliative and End of Life Care: A national framework for local action 2015-2020.

[36] Ziegler LE, Craigs CL, West RM, Carder P, Hurlow A, Millares-Martin P, et al. Is palliative care support associated with better quality end-of-life care indicators for patients with advanced cancer? A retrospective cohort study. BMJ Open. 2018;8. 10.1136/bmjopen-2017-018284. 10.1136/bmjopen-2017-018284 PMC5829853 29386222

[37] Lloyd A, Kendall M, Carduff E, Cavers D, Kimbell B, Murray SA (2016). Why do older people get less palliative care than younger people?. Eur J Palliat Care.

[38] Hazra NC, Rudisill C, Gulliford MC (2018). Determinants of health care costs in the senior elderly: age, comorbidity, impairment, or proximity to death?. Eur J Health Econ.

